# Different Covalent Immobilizations Modulate Lipase Activities of *Hypocrea pseudokoningii*

**DOI:** 10.3390/molecules22091448

**Published:** 2017-09-04

**Authors:** Marita G. Pereira, Susana Velasco-Lozano, Sonia Moreno-Perez, Aline M. Polizeli, Paulo R. Heinen, Fernanda D. A. Facchini, Ana C. Vici, Mariana Cereia, Benevides C. Pessela, Gloria Fernandez-Lorente, Jose M. Guisan, João A. Jorge, Maria de Lourdes T. M. Polizeli

**Affiliations:** 1 Departamento de Biologia, Faculdade de Filosofia, Ciências e Letras de Ribeirão Preto, Universidade de São Paulo, Av. Bandeirantes, 3900, Ribeirão Preto-SP 14040-901, Brazil; maritagimenez@hotmail.com (M.G.P.); aline.polizeli@nutreco.com (A.M.P.); acvici@usp.br (A.C.V.); macereia@ffclrp.usp.br (M.C.); joajorge@ffclrp.usp.br (J.A.J.); 2 Heterogeneous Biocatalysis Group, CIC Biomagune, Parque Tecnológico de San Sebastián Edificio Empresarial “C”, Paseo Miramón 182, 20009 Donostia-San Sebastián Guipúzcoa, Spain; svelasco@cicbiomagune.es; 3 Departamento de Biotecnología y Microbiología de los Alimentos, Instituto de Ciências de la Alimentación, CIAL-CSIC, Calle Nicolás Cabrera 9, Campus UAM, Cantoblanco, 28049 Madrid, Spain; sonia.moreno@csic.es (S.M.-P.); b.pessela@csic.es (B.C.P.); gflorente@ifi.csic.es (G.F.-L.); 4 Departamento de Biocatálisis, Instituto de Catálisis y Petroleoquímica, CSIC, Campus UAM, Cantoblanco, 28049 Madrid, Spain; jmguisan@icp.csic.es; 5 Departamento de Bioquímica e Imunologia, Faculdade de Medicina de Ribeirão Preto, Universidade de São Paulo, Ribeirão Preto-SP 14040-900, Brazil; prheinen_1613@hotmail.com (P.R.H.); fer_facchini@yahoo.com.br (F.D.A.F.)

**Keywords:** enzyme immobilization, lipase activity modulation, stability, hydrolysis of oils, *Hypocrea pseudokoningii*

## Abstract

Enzyme immobilization can promote several advantages for their industrial application. In this work, a lipase from *Hypocrea pseudokoningii* was efficiently linked to four chemical supports: agarose activated with cyanogen bromide (CNBr), glyoxyl-agarose (GX), MANAE-agarose activated with glutaraldehyde (GA) and GA-crosslinked with glutaraldehyde. Results showed a more stable lipase with both the GA-crosslinked and GA derivatives, compared to the control (CNBr), at 50 °C, 60 °C and 70 °C. Moreover, all derivatives were stabilized when incubated with organic solvents at 50%, such as ethanol, methanol, *n*-propanol and cyclohexane. Furthermore, lipase was highly activated (4-fold) in the presence of cyclohexane. GA-crosslinked and GA derivatives were more stable than the CNBr one in the presence of organic solvents. All derivatives were able to hydrolyze sardine, açaí (*Euterpe oleracea*), cotton seed and grape seed oils. However, during the hydrolysis of sardine oil, GX derivative showed to be 2.3-fold more selectivity (eicosapentaenoic acid (EPA)/docosahexaenoic acid (DHA) ratio) than the control. Additionally, the types of immobilization interfered with the lipase enantiomeric preference. Unlike the control, the other three derivatives preferably hydrolyzed the *R*-isomer of 2-hydroxy-4-phenylbutanoic acid ethyl ester and the *S*-isomer of 1-phenylethanol acetate racemic mixtures. On the other hand, GX and CNBr derivatives preferably hydrolyzed the *S*-isomer of butyryl-2-phenylacetic acid racemic mixture while the GA and GA-crosslink derivatives preferably hydrolyzed the *R*-isomer. However, all derivatives, including the control, preferably hydrolyzed the methyl mandelate *S*-isomer. Moreover, the derivatives could be used for eight consecutive cycles retaining more than 50% of their residual activity. This work shows the importance of immobilization as a tool to increase the lipase stability to temperature and organic solvents, thus enabling the possibility of their application at large scale processes.

## 1. Introduction

Lipases are α/β hydrolases which catalyze the hydrolysis of acylglycerols to release fatty acids and glycerol. These enzymes can be divided into two main groups: (i) carboxylesterases (EC 3.1.1.1) and (ii) “true” lipases or triacylglycerol lipases (EC 3.1.1.3), which differ in several biochemical features [1,2]. After determining the three-dimensional structure of this latter type, the presence of a “lid” was verified. This lid is known as a protein surface loop that covers the enzyme active site and is directly related to the “interfacial activation”. This promotes a conformational change in the enzyme structure causing the movement of the lid which is in contact with this interface, exposing the active site and activating the “true” lipases. Its activity should sharply increase as soon as it is in contact with the triglyceride substrate [2,3,4].

Lipases are ubiquitous in Nature and they are produced by plants [5], microorganisms [6] and mammals [7]. Lipases from microorganisms are widely used for biotechnological applications, because they are usually more stable than the ones obtained from animals and plants, which makes their production more convenient, safer, easier, less expensive and they are generally more stable in organic solvents. Moreover, lipases from this source show versatility in their properties with respect to the enzymatic activity and substrate specificity [2,8,9]. Fungi are able to secrete large amounts of enzymes in the extracellular environment. Therefore, microbial lipases from fungi have been widely used to produce lipases in the last decades [9].

The use of lipases in industry has continuously grown, especially in industries such as food, dairy, pharmaceuticals, agrochemicals, detergents, oleo-chemicals, tea industries, cosmetics, leather, as well as in several bioremediation processes [8,9,10]. The most recent application of lipases is in the biosensor industry [11,12]. Manufacturers in many other important industries, including health care, pharmaceuticals [13] and chemicals [14], are increasingly taking advantage of Nature’s amazing catalysts. For the last few years, enzymes have widely been used in the production of biofuels such as biodiesel [15]. One of the best uses of lipases in modern life is in the treatment of wastes in general [16], especially in the treatment of solid wastes [17] and in the purification of waste water [18]. In some cases, industrial applications of enzymes in organic solvents are also developed [19]. Moreover, microbial enzymes, including lipases, can be produced from renewable raw materials. In addition, the mild operating conditions of enzymatic processes mean that they can be operated in relatively simple and totally controlled equipment. However, all these desirable characteristics of enzymes, and their widespread industrial applications are often hampered by their lack of long-term operational stability and shelf-storage life, low thermal and chemical stability, and by their cumbersome recovery and re-use [12,20].

In order to enhance the economy of biocatalytic processes it is possible to perform enzyme immobilization [21]. Improvements in current immobilization strategies have progressed by using supports that increase the effectiveness and stability of the connection through multipoint attachments. Novel methods of enzyme self-immobilization have been developed (CLEC, CLEA, Spherezyme), as well as carrier materials (Dendrispheres), encapsulation (PEI microspheres), and entrapment. Apart from retention, recovery and stabilization, other advantages of enzyme immobilization have emerged, such as enhanced enzyme activity, modification of substrate selectivity and enantioselectivity, and multi-enzyme reactions. Protein immobilization commonly alters the natural molecular environment of the enzymes, and often affects their catalytic activity due to modification of the substrate’s accessibility to the active center, decreased enzyme molecular mobility and changes in its conformational integrity [22]. These advances promise to enhance the role of enzyme immobilization in industry, opening the way to novel applications [23].

Immobilization allows re-using the enzyme for an extended period and enables easier separation of the catalyst from the product. Additionally, immobilization improves many properties of enzymes such as their performance in organic solvents, pH tolerance, and heat stability. The increase of the structural rigidity of the protein and stabilization of multimeric enzymes prevent dissociation-related to inactivation [21]. Immobilization is attractive for lipases because, besides all the advantages common to other proteins, it can also considerably increase the catalytic activity, probably due to conformational changes that better expose the active site of the enzyme [24].

Supports activated with glutaraldehyde or primary amino groups can be used for enzyme immobilization [25]. Among them, chitin and chitosan are the most important functional materials for immobilization. They offer biocompatibility, biodegradability, nontoxicity, physiological inertness, antibacterial properties, heavy metal ion chelation, gel forming properties and hydrophilicity, and remarkable affinity to proteins [26]. The cationic nature of their surface allows the rapid ionic attachment of the protein at low-ionic strengths [27]. Besides, there are two different possibilities: (1) direct covalent-attachment of the enzyme on glutaraldehyde-activated supports, and (2) adsorption of the proteins on amino-activated supports followed by glutaraldehyde cross-linking of both the enzyme and the support [28]. In this work, these two different attaching chemistries are employed for the stabilization of the studied lipase. The immobilization of the lipase from *Hypocrea pseudokoningii*, which has been scarcely studied, is presented, thus contributing with its exploitable catalytic properties. This work also shows an enzymatic biocatalyst highly stable in organic solvents, high temperatures and under denaturing conditions, providing many important contributions for the future application of this enzyme.

## 2. Results and Discussion

### 2.1. Immobilization of Enzymes on Different Supports

**Figure 1:**
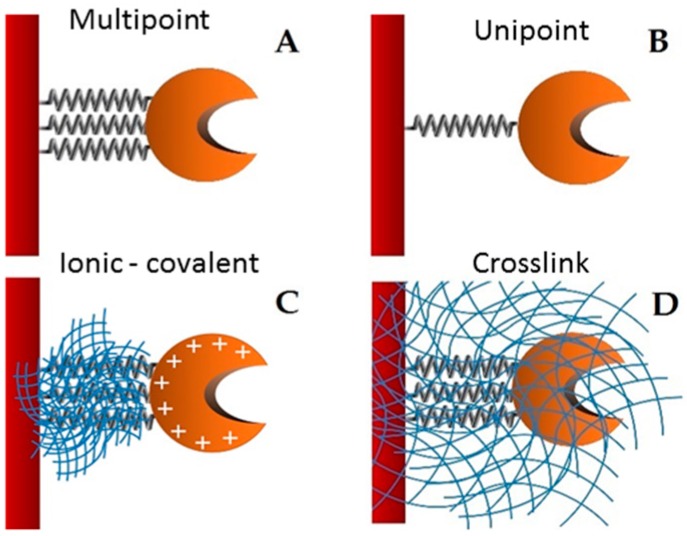
Schematic representation of the derivatives (enzyme immobilized + support) used in the present work. (**A**) GX; (**B**) CNBr (control); (**C**) GA; (**D**) GA-crosslink.

*H. pseudokoningii* lipase was immobilized in four covalent supports: Gglyoxyl—agarose (GX, Figure 1A), cyanogen bromide—agarose (CNBr-activated, Figure 1B), MANAE—agarose activated with glutaraldehyde (GA, Figure 1C) and crosslinked prepared from a GA support (GA-crosslink, Figure 1D).

The lipase was rapidly and fully immobilized on all covalent supports, but there were differences on the activation among the supports. The lipase was almost totally inhibited on GX support, while, on GA and GA-crosslink it was activated 1.35-fold and 2.36-fold, respectively (Table 1). Supports activated with glutaraldehyde (GA) or the treatment of the adsorbed enzymes with glutaraldehyde (GA-crosslink) formed covalent bounds between the enzyme and the support. The lipase was strongly attached to the support because even in the presence of 3 M NaCl it was not desorbed (data not shown). Besides, GX-support also allowed an intense multi-point binding of the enzyme. However, the alkaline conditions required during the immobilization procedure on GX-support led to the partial inactivation of the biocatalyst. GX immobilization could be advantageous because this process makes the enzyme structure more rigid due to the formation of multipoint covalent bonds and it makes the enzyme more resistant to the presence of denaturing agents. In contrast, the lipase immobilized on GA and GA-crosslink was activated during the immobilization. This activation may have been caused by the amount of crosslinking of glutaraldehyde, the enzyme and the support [25].

### 2.2. Effect on the Stability of Different Enzyme Preparations

**Figure 2:**
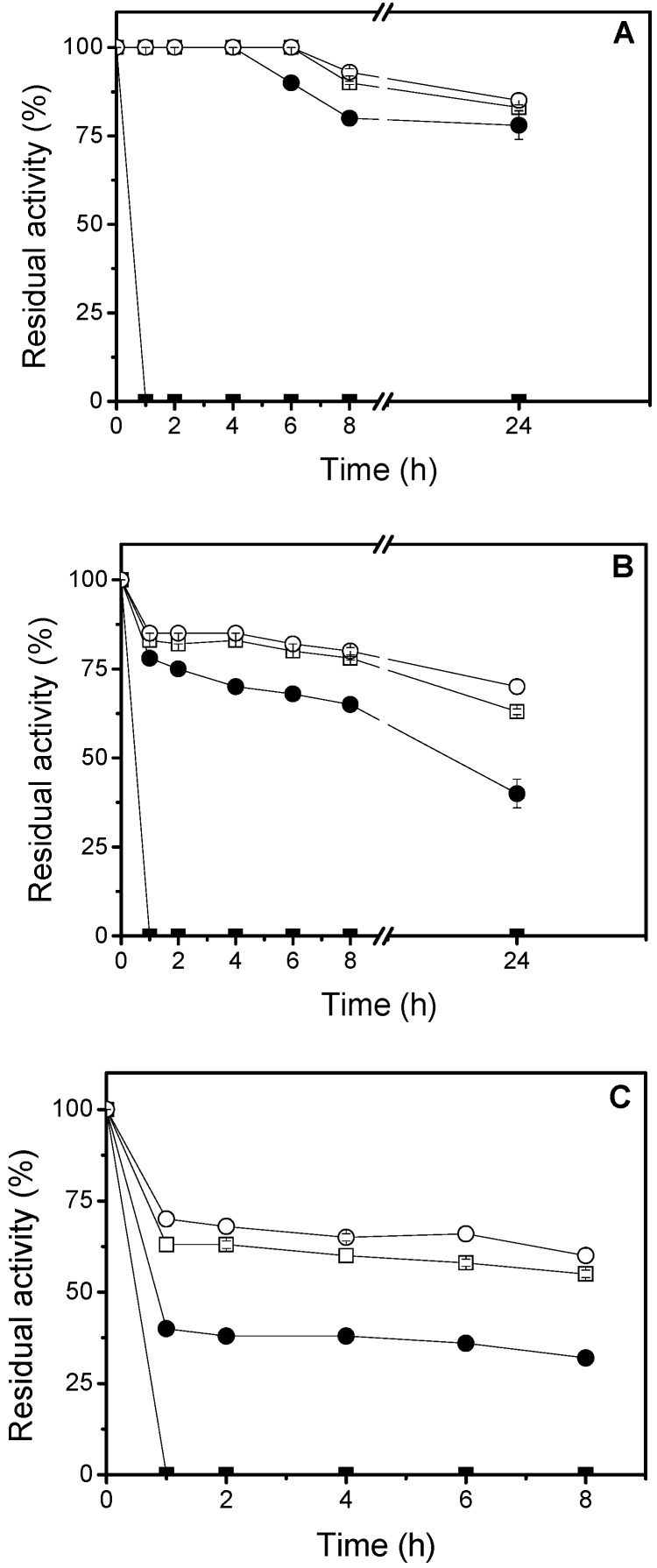
Thermal stability of lipases adsorbed on covalent supports. (**A**) 50 °C; (**B**) 60 °C; (**C**) 70 °C. Symbols: ■ CNBr derivative; ● GA-crosslink derivative; □ GX derivative; ○ GA derivative.

Thermostability of the immobilized lipase on different covalent supports was compared at 50 °C, 60 °C and 70 °C. Figure 2 shows that the lipase immobilized on CNBr was rapidly inactivated at all temperatures tested, while all other derivatives remained 100% active up to 4 h of incubation in aqueous media at 50 °C.

After that, they maintained over 80% of the residual activity, up to 24 h (Figure 2A). At 60 °C (Figure 2B), GA-crosslink derivative reached half-life after 8 h and still retained about 38% of the residual activity for up to 24 h; while, GA and GX derivatives, within 24 h of assay, still maintained approximately 74% and 63% of the residual activity, respectively. At 70 °C (Figure 2C), the activities of GA-crosslink, GX and GA derivatives remained constant after the first 2 h, around 38%, 63% and 70% of the residual activity, respectively.

All other examples were more stable than the CNBr derivative, which is commonly used as a control because it simulates the activity of native enzymes when compared to the ones immobilized on other supports [29]. Pereira et al. [29] also reported that the ionic and hydrophobic immobilization processes protected the enzyme against heat inactivation. This stabilization is related to the formation of multiple linkages between the enzyme and the support, providing higher rigidity to its tertiary structure and less distortion on the catalytic site [27], preventing protein denaturation at high temperatures. GX and GA supports were also applied in the thermal stabilization of other lipases [30,31]. A very interesting fact observed, regarding GX derivative (Figure 2) was its thermostability, once it was partially inactivated during the immobilization process (Table 1). This fact has corroborated with our observations that the support on which the enzyme is more activated, does not always make it more stable.

### 2.3. Effect of Organic Solvents on the Stability of the Immobilized Lipase

**Figure 3:**
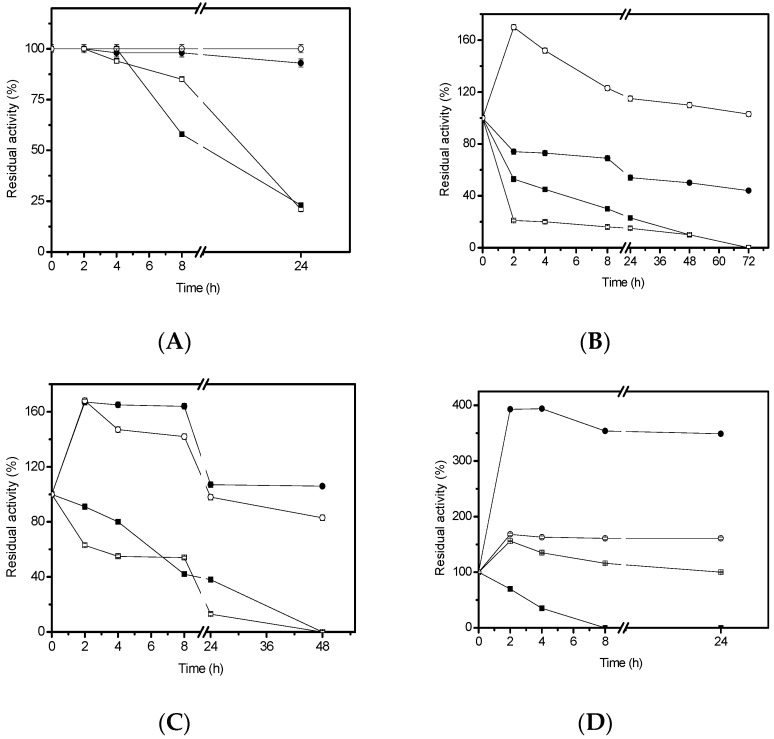
Stability of derivatives in organic solvents. (**A**) ethanol; (**B**) methanol; (**C**) *n*-propanol; (**D**) cyclohexane. Symbol: ■ CNBr derivative; ● GA-crosslink derivative; □ GX derivative; ○ GA derivative.

The stability of lipase derivatives from *H. pseudokoningii* was investigated in the presence of organic solvents at a final concentration of 50%, at 4 °C. GA-crosslink and GA derivatives were completely stable in ethanol for up to 24 h (Figure 3A). CNBr and GX derivatives were stable only for 4 h, decreasing their activities within 24 h in the presence of ethanol (Figure 3A) or showing half-lives of 2 h with methanol (Figure 3B). On the other hand, GA-crosslink derivative reached a half-life with 24 h. Surprisingly, the GA derivative had a 1.6-fold activation in the first 2 h, and then 100% of the residual activity remained for 72 h. When the derivatives were incubated in *n*-propanol (Figure 3C), CNBr and GX derivatives showed half-lives of 7 and 8 h, respectively. GA-crosslink and GA derivatives showed 1.6-fold activation and maintained about 100% and 80% of the residual activity for up to 48 h, respectively. In the presence of cyclohexane (Figure 3D), all derivatives showed excellent stability, except the CNBr one that showed a half-life of 3 h and was completely inactivated after 8 h of incubation. GX derivative underwent 50% activation (2 h), and retained 100% of residual activity for up to 24 h. GA derivative presented an activation of approximately 1.8-fold and maintained this activation for 24 h. GA-crosslink derivative showed about 4-fold activation with 24 h of incubation.

There are numerous advantages to employing enzymes as catalysts in organic solvents or aqueous solutions containing organic solvents. A few natural enzymes which are stable in the presence of organic solvents have been discovered [32]. However, almost all lipases are easily denatured and inactivated in the presence of organic solvents. Therefore, the immobilization technique improves enzyme stability in the presence of organic solvents. It is known that the stability in organic environment is an important property to lipases, giving them interesting features to be applied in organic synthesis. The immobilized lipase from *H. pseudokoningii* not only presented excellent thermostability as it was also highly stable in the presence of organic solvents at 25 °C. The effect of the organic medium depends on the nature of both, enzyme and solvents [33]. In this study, the higher stabilization was attained in the presence of cyclohexane. This hyper-activation effect is probably related to an increase in substrate solubility. This effect is observed in GA derivative when it was tested mainly in the presence of cyclohexane. Maybe this occurred because of the increase in the solubility of substrate and the way the enzyme was immobilized. Presumably, the immobilization did not occur through the lid which recovers the catalytic site, so the lid probably has a mobile conformation, leading to an increase of the activity to the entrance of substrate. The enzyme activity in the presence of organic solvents also depends on their concentration and the nature of the enzymes [34,35,36]. Stabilization of lipases towards denaturant solvents has also been reported in other works [29,37].

### 2.4. Hydrolysis of Oils

The derivatives were tested in the hydrolysis of sardine oil and it was observed that all of them were able to hydrolyze this oil (Table 2). Different activities among the distinct derivatives were observed, in relation to the immobilization effect on enzyme activity and selectivity for eicosapentaenoic acid (EPA) and docosahexaenoic acid (DHA). All four biocatalysts preferentially hydrolyzed EPA more than DHA. Besides, in comparison to CNBr derivative, GA derivative increased its selectivity towards EPA.

EPA and DHA are commonly obtained as byproducts of fish oil processing [38]. These oils contain about 30% of omega-3 PUFAs in combination with different fatty acids, mainly in the form of triglycerides [38]. However, their purification represents a main drawback since their structures are very similar. In order to overcome such challenge, lipases have been successfully applied, since these enzymes exhibit great EPA/DHA selectivity [39]. Therefore, the application of the prepared derivatives of the studied lipase in the hydrolysis of sardine oil was an interesting approach. Besides, improved activity and EPA/DHA selectivity was attained with GA derivative in comparison to CNBr (Table 2). Other authors also reported improved EPA/DHA selectivity by the immobilization of lipases [40]. Fernandez-Lorente et al. [40] showed the release of omega-3 fatty acids by the mild enzymatic hydrolysis of sardine oil using lipases physically adsorbed on hydrophobic porous supports. These immobilized lipases could only hydrolyze oil molecules partitioned into the aqueous phase of a biphasic reaction system. In general, hydrophobic lipase derivatives were found to be more active and more selective for the release of EPA than CNBr-activated. The most interesting biocatalyst was the hydrophobic derivative of *Yarrowia lipolytica* lipase, which was found to be sevenfold more active and tenfold more selective than CNBr-activated one, like in this work. Fernandez-Lorente also showed that the most active (but non-selective) derivative was the hydrophobic derivative of *Pseudomonas fluorescens* lipase. This happened because hydrophobic supports promote the interfacial activation of lipases, similar to the interaction promoted by oil drops on soluble enzymes [40].

**Figure 4:**
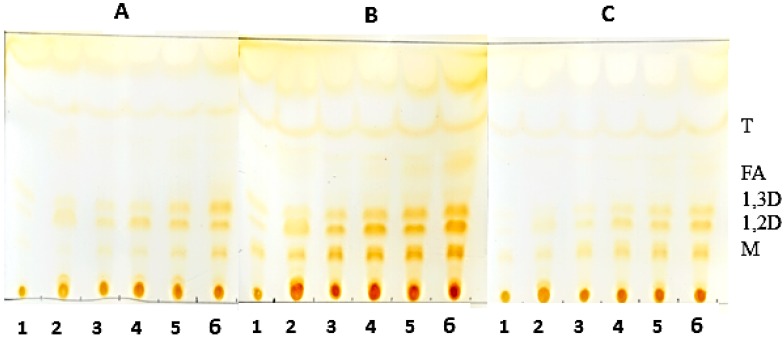
Thin-layer chromatography of the hydrolysis products of oils catalyzed by lipase derivatives. (**A**) Açaí oil; (**B**) Cotton seed oil; (**C**) Grape seed oil. 1: Reaction without enzyme; 2: free lipase; 3: CNBr derivative; 4: GA derivative; 5: GA-crosslink derivative; 6: GX derivative. **T**—triacylglycerol, **FA**—fatty acids, **D**—diacylglycerol, **M**—monoacylglycerol.

The four derivatives were also tested for the hydrolysis of other oils: açaí, cotton seed, and grape seed oils. Although all three oils were hydrolyzed by all derivatives, different activities were observed for each of them (Table 3). GA derivative presented the highest activity on açaí oil, followed by CNBr, GA-crosslink and GX derivatives. On cotton seed oil, the higher activity was reached with CNBr, followed by GA-crosslink, GA and GX derivatives. Grape seed oil presented higher activity values, up to 13-fold. The highest activity was reached with GA-crosslink derivative and the lowest with GX; however, CNBr and GA derivatives had similar activities (Table 3). Results from thin-layer chromatography (TLC) analysis corroborate the ones observed for the hydrolysis products of oils catalyzed by the lipase derivatives (Figure 4). All derivatives had high activity on oils, showing the generation of diacylglycerol, monoacylglycerol, and fatty acids.

Differences in activity were observed, indicating that the immobilization protocol influenced this parameter as well as the enzyme specificity. The oils used in the present work have important nutritional and medicinal properties and an interesting feature is that they have different compositions [41], thus allowing the attainment of different fatty acids. Açaí oil consists mainly of oleic acid (53%), followed by palmitic acid (26%), while cotton seed oil has three main constituents: linoleic acid is present at a higher concentration (46%), followed by oleic acid (30%) and palmitic acid (24%). Grape seed oil is mainly composed by linoleic acid (60%), followed by oleic acid (12%). The different derivatives prepared in this work preferentially hydrolyzed fatty acids with 18 and 16-carbons.

### 2.5. Enantioselectivity of Different Derivatives

When hydrolyzing *racemic*-2-hydroxy-4-phenylbutanoic acid ethyl ester (*rac*-HPBE) the CNBr derivative hydrolyzed the *S*-HPBE isomer faster than the *R* isomer (Table 4), determining the enantioselectivity of the derivatives, but the observed stereoselectivity was too low for practical applications (*E* = 3.0). The difference of enantiomeric excess was 2.5-fold higher on GA derivative (*ee* = 6.4) compared to CNBr, but the stereoselectivity was similar (*E* = 3). CNBr derivative showed an activity of 7.41 U/mg and GA of 13.33 U/mg. GX and GA-crosslink derivatives showed higher specific activity 21 and 21.88 U/mg, respectively, but the stereoselectivity was the same (*E* = 5). The difference of enantiomeric excess was 10.5-fold higher on GX derivative (*ee* = 27.2) and 13.2-fold higher on GA-crosslink (*ee* = 34.3) compared to CNBr.

The enantioselectivity study of mixture *racemic*-butyryl-2-phenylacetic acid (*rac*-BPA) was conducted with the derivatives, which showed differences in their enantiomeric preference. In this reaction, a large difference among the derivatives occurred (Table 5) as the specific activity of the GX derivative was 87-fold higher than the CNBr one. The stereoselectivity was also higher for GX (*E* = 4) than for CNBr (*E* = 3). The GX derivative showed an enantiomeric excess (*ee*) 4.1-fold higher than CNBr, indicating that the immobilization enhanced lipase selectivity. An increase in selectivity occurred with the GA and GA-crosslink derivatives, which had enantiomeric excesses of 11.1% and 10.2%, respectively.

It was also possible to observe a difference between their specific activities. GA derivative showed specific activity 128-fold higher than CNBr (0.06 U/mg), whereas the GA-crosslink derivative presented specific activity 39-fold higher than CNBr. Both the GA and GA-crosslink derivatives showed stereoselectivity of *E* = 5. Another extremely interesting fact is that immobilization causes changes in enantiomeric preference. The CNBr and GX derivatives showed preference for the *S* isomer, while the GA-crosslink and GA derivatives showed an enantiomeric preference for the *R* isomer.

The derivatives were also tested in the presence of methyl mandelate (*rac*-MEMA) (Table 6). All four derivatives showed enantiomeric preference for the *S*-isomer. The CNBr derivative showed a lower specific activity and enantiomeric excess than other covalent derivatives. The enantiomeric rate was 2-fold higher for GA derivative than CNBr, thus showing that the immobilization could improve the stereoselectivity of the biocatalysts.

The activity in the presence of racemic-1-phenylethanol acetate (*rac*-AFE) showed an inversion in enantiomeric preference (Table 7). The CNBr derivative preferred the *R*-isomer, while other derivatives preferred the *S*-isomer. The CNBr derivative produced higher *ee* than the GX and GA-crosslink derivatives (4.6, 1.3 and 1.6, respectively), and while the CNBr and GX derivatives showed *E* = 4, the *E* value for GA and GA-crosslink was *E* = 3. The specific activities were 5.9, 3.1, 5.3, 7.3 U/mg for CNBr, GX, GA and GA-crosslink derivatives, respectively. Enantioselectivity is an exquisite feature of lipases making them powerful tools in the preparation of optically pure compounds. Therefore, the prepared biocatalysts were also tested in the hydrolytic resolution of several carboxylic acids and alcohols since they are important building blocks in the preparation of valuable pharmaceutical compounds.

This work suggests that the properties of a lipase may be strongly modulated via immobilization, which is altered by the rigidity and the kind of link. Thus, the same lipase immobilized on different supports, having different rigidity and microenvironment, exhibited very different catalytic properties [43]. Changes can be obtained simply by using different techniques. In fact, the same lipase immobilized on different supports behave as different lipases.

All derivatives showed a wide diversity in relation to the predilection for *R* and *S* forms, indicating that the immobilization caused changes in the enantiomeric preferences. These derivatives are examples of how the immobilization can cause some distortion to the enzyme structure keeping its similar orientation, and may exert an unexpected effect on enzyme specificity, improving its activity in the presence of some substrates or decreasing it with others. [44]. However, with all the racemic substrates tested, *ee* values were very low for any practical application. Nevertheless, immobilization on GA support caused the inversion of the enantiopreference when *rac*-methyl mandelate, *rac*-HPBE and *rac*-AFE were hydrolyzed.

### 2.6. Reuse and Operational Stability of the Enzyme

**Figure 5:**
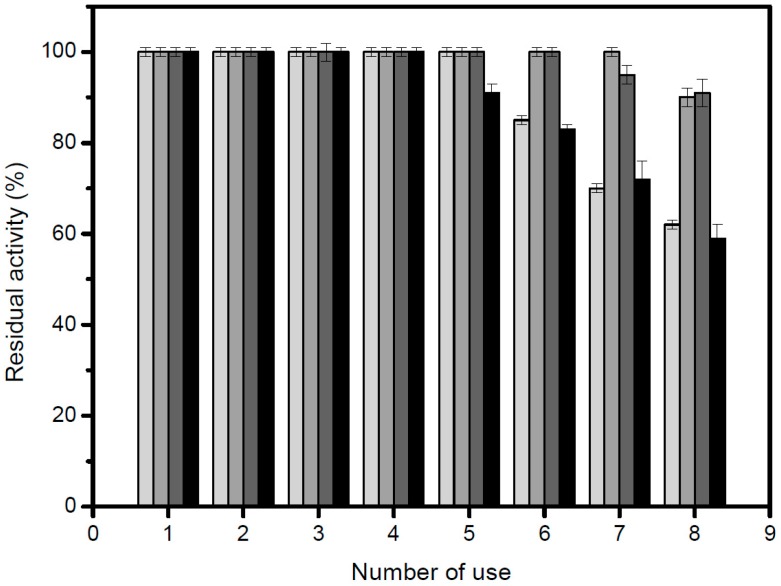
Operational stability of the lipase derivatives in repeated use conditions. Legends: light gray color, GX derivative; gray color, GA-crosslink derivative; dark gray color, GA derivative; black color, CNBr derivative.

Reusability studies of the derivatives were carried out using the recovered enzyme for subsequent cycles in the reaction with *p*-nitrophenyl palmitate. After each cycle, the derivatives were filtered, washed with fresh buffer and allowed to drain before reuse. Operational stability of the derivatives showed residual activity of 100% up to the 5th cycle (Figure 5). After that, up to the 8th cycle, the derivatives showed more than 50% of the residual activity. The best derivatives were GA-crosslink and GA. Nevertheless, all derivatives tested showed good results concerning the possibility of reuse. This result was expected, since the covalent immobilization stabilizes the enzyme, avoiding denaturation. In fact, this stabilization occurred due to the intense linking between the enzyme and the support thus, promoting high stabilization [25,45].

## 3. Material and Methods

### 3.1. Microorganism, Culture Conditions and Lipase Production

*Hypocrea pseudokoningii* was maintained at 30 °C in slants of 4% PDA. Conidia from 7-day old culture were inoculated into 50 mL of Adams liquid medium [46] (final concentration of 10^5^ spores/mL). Cultures were incubated in a rotational shaker (110 rpm), for 96 h, at 30 °C [6]. After that, the mycelia were separated from the extracellular medium using vacuum filtration on no. 1 Whatman filter paper, and the crude filtrate was used as a source of extracellular lipase activity.

### 3.2. Measurement of Lipase Activity and Protein

#### 3.2.1. Hydrolysis of *p*-Nitrophenyl Butyrate (*p*NPB)

This assay was performed by measuring the increase in the absorbance at 348 nm produced by the release of *p*-nitrophenol in the hydrolysis of 0.4 mM *p*NPB in 25 mM sodium phosphate buffer at pH 7 and 25 °C, using a spectrophotometer equipped with a thermostatized chamber and continuous magnetic stirring to keep the immobilized enzyme homogenously suspended. The beginning of the reaction occurred with 0.1 mL of lipase solution or a suspension added to 2.5 mL of substrate solution. The presence of solids during the assay only produced a marginal increase in the noise of readings and did not affect the measurement of absorbance.

#### 3.2.2. Hydrolysis of *p*-Nitrophenyl Palmitate (*p*NPP)

This assay was performed by measuring the increase in the absorbance at 400 nm produced by the release of *p*-nitrophenol in the hydrolysis of 0.4 mM *p*NPP in 25 mM acetate sodium buffer at pH 6 and 45 °C. For that, it was used a spectrophotometer equipped with a thermostatized chamber and continuous magnetic stirring to keep the immobilized enzyme homogenously suspended. The beginning of the reaction occurred with 0.1 mL of lipase solution or a suspension added to 2.5 mL of substrate solution. The presence of solids during the assay only produced a marginal increase in the noise of readings and did not affect the measurement of the absorbance. One unit (U) of enzyme activity was defined as that catalyzing the conversion of 1 μmol of substrate (or the formation of 1 μmol of product) in 1 min, in the assay conditions. Proteins were measured according to Bradford method [47], using bovine serum albumin as standard and expressed by mg protein/mL. Specific activity (SA) was expressed as Units/mg protein.

### 3.3. Purification of Lipase from Hypocrea Pseudokoningii

Lipase from *H. pseudokoningii* was purified using immobilization on Octyl Sepharose support in 5 mM sodium phosphate buffer solution, pH 7, at 4 °C [29]. After the immobilization, adsorbed lipase derivatives were extensively washed with distilled water and the purity was determined. Afterwards, the immobilized enzyme was desorbed with 2% Triton X-100, resulting in a purified enzyme extract.

### 3.4. Support Preparation and Enzyme Immobilization

Glyoxyl agarose gel was prepared according to Guisan [48]. Monoaminoethyl-*N*-ethylagarose (MANAE) was synthesized according to Fernandez-Lafuente et al. [49] MANAE activated with glutaraldehyde and crosslinked were prepared according to Betancor [28]. The biocatalyst obtained by the immobilization through this support was named GA-crosslink.

A mass of 1 g of covalent support (glyoxyl—agarose and MANAE activated with glutaraldehyde) was added to 10 mL of the purified enzyme solution (with a maximum enzyme activity of 5 U/mL and 0.25 mg/mL) in different buffers according to each immobilization. The immobilization occurred overnight. After this period, the derivative (support containing the enzyme) was separated of the supernatant by filtration. The biocatalyst immobilization obtained from lipase plus glyoxyl-agarose and MANAE activated with glutaraldehyde were called GX and GA derivatives, respectively. After enzyme immobilization, linkages between glyoxyl and amine groups were reduced with 1.0 mg/mL sodium borohydride for 30 min at room temperature. The derivatives were washed with distilled water and maintained at 4 °C.

Samples of the supernatants and suspensions were periodically withdrawn, and the enzyme activity was measured. The immobilization was considered completed when no activity was detected in the supernatant. Sodium phosphate buffer (25 mM) at pH 7.0 and 8.0, at 25 °C as well as 25 mM sodium bicarbonate buffer at pH 10.5, 4 °C, were used for lipase immobilization in MANAE-crosslink, MANAE activated with glutaraldehyde, and glyoxyl agarose, respectively. The yield of immobilization was considered to be the rate between the activities in the supernatant compared to the activity in the blank of soluble enzyme (initial solution). In all cases, the activity of the blank was 100% during the immobilization process. Activity recovery was calculated through the ratio of the activity in the derivative after the immobilization process and the initial activity of the offered enzyme.

### 3.5. Immobilization of Lipases on Cyanogen Bromide (CNBr-Activated) Support

This derivative was prepared using the Pharmacia^®^ protocol at pH 7.0. The immobilization should occur via the most reactive group: the amino terminal [47,50]. One gram of CNBr-activated support was added to a solution of 10 mL of the pure lipase (0.25 mg/mL) in the present of 25 mM sodium phosphate buffer containing 2% (*v*/*v*) Triton X-100 at pH 7.0, 4 °C. After 15 min, 100% of the lipase became immobilized. The enzyme immobilization on CNBr was ended by blocking the amine reactive groups of the support in the presence of ethanolamine 1 M at pH 8.0. After 2 h, the immobilized preparation was washed with abundant water.

### 3.6. Thermal Stability Studies

In order to study the thermal stability, 4.68 U/g of the immobilized lipase were used. The inactivation was carried out at 50 °C, 60 °C and 70 °C, in 25 mM sodium phosphate buffer, at pH 7.0. At different times, samples were withdrawn and their activities were tested as previously described. The remaining activity was calculated as the ratio between the activity at a given time and the activity at time “zero” of incubation. Half-lives were calculated from the observed inactivation courses.

### 3.7. Organic Solvents Stability Studies

In order to study the lipase stability in organic solvents, 4.68 U/g of the immobilized lipase were incubated with 50% (*v*/*v*) ethanol, methanol, *n*-propanol, or cyclohexane. The inactivation was carried out in 25 mM sodium phosphate buffer at pH 7.0 and room temperature. At different times, samples were withdrawn and their activities were tested. The remaining activity was calculated between the activity at a given time and activity at time zero of incubation.

### 3.8. Hydrolysis of Oils

The hydrolysis of sardine oil was performed in a water-organic solvent two-phase system as described [51] (4.5 mL of cyclohexane, 5 mL of 0.1 M Tris buffer pH 6.0 and 0.5 mL of sardine oil). The reaction was initiated by adding 0.4 g of lipase derivatives, and the mixture was mechanically stirred at 600 rpm for 24 h, at 25 °C. A volume of 100 μL of the sample present in the organic phase were removed and mixed with 400 μL acetonitrile. Reactants and products were analyzed by HPLC (SP 100 coupled with a SP 8450 UV detector, Spectra Physics, Santa Clara, CA, USA) using a Kromasil C8 column (150 × 4.6 mm). Products were eluted at a flow rate of 1.5 mL/min with acetonitrile/water/acetic acid (70:30:0.1 *v*:*v*:*v*) and pH 3.0. The UV detection was performed at 215 nm. The retention times for the unsaturated fatty acids were 9.4 min for EPA and 13.5 min for DHA.

The hydrolysis of açaí (*Euterpe oleracea*), grape seed and cotton seed oils was performed in 25 mM sodium acetate buffer, pH 5.0, using an automatic titrator. The reaction was performed as follows: 6.0 mL of oil, 8.0 mL of 25 mM sodium acetate buffer, and 0.5 mL of Triton X-100. The reaction was initiated by adding 300 mg of the immobilized lipase, and the mixture was mechanically stirred at 250 rpm, 45 °C, for 16 h. The reaction was interrupted by the addition of a solution constituted by ethanol: acetone (1:1). The total unit (Total U) is defined as the amount of enzyme required to release one μmol of fatty acid in one minute (μmols/min), considering the total volume of the reaction.

### 3.9. Thin-Layer Chromatography of the Hydrolysis Product of Oils Catalyzed by Derivatives

Analysis of the hydrolysis products was obtained by thin layer chromatography (TLC) on silica plates (DC-Alufolien Kieselgel 60 without fluorescence indicator, Merck^®^, Darmstadt, Germany). The reaction mixture consisted of 2.0 mL of oil, 300 mg of derivative, 0.3 mL of Triton X-100 and, 0.7 mL 500 mM MES buffer, pH 6.0. Incubation of the mixture was carried out at 45 °C under constant agitation. Aliquots were removed and 5.0 μL were applied to a silica plate. The run was carried out twice with a mixture of hexane-ethyl acetate-acetic acid (90:10:1). The visualization of the hydrolysis products was performed by exposure to iodine vapor until the appearance of bands corresponding to the products of triglyceride degradation.

### 3.10. Enzymatic Hydrolysis of Racemic Mixtures

The activities of different preparations of lipase from *H. pseudokoningii* on the hydrolysis of racemic mixtures were investigated by adding the enzyme preparations (0.3 g) to 3.0 mL of 5.0 mM (*R*,*S*)-substrate under mechanical stirring. At different times, the degree of hydrolysis was measured by reverse-phase HPLC (Spectra Physics SP 100 coupled with a Spectra Physics SP 8450UV detector) on a Kromasil C18 (25 cm × 0.4 cm) column supplied by Analysis Vinicos (Tomelloso, Spain) using the corresponding mobile phase and flow rates listed in Section 3.11 below. The elution was monitored by recording the absorbance at 254 nm. Triplicates (at least) of each assay were made and experimental error was never higher than 5%.

### 3.11. Mobile Phases

#### Enzymatic Hydrolysis of Racemic-2-hydroxy-4-phenylbutanoic Acid Ethyl Ester (*rac*-HPBE)

The mobile phase consisted of acetonitrile (40%) and 10 mM ammonium phosphate buffer (60%) at pH 2.9 and a flow rate of 1.0 mL/min.

#### Enzymatic Hydrolysis of Racemic Butyryl-2-phenylacetic Acid (*rac*-BPA)

The mobile phase consisted of acetonitrile (35%) and 10 mM ammonium phosphate buffer (65%) at pH 2.5 and a flow rate of 1.5 mL/min.

#### Enzymatic Hydrolysis of Racemic Methyl Mandelate (*rac*-MEMA)

The mobile phase consisted of acetonitrile (40%) and 10 mM ammonium phosphate buffer (60%) at pH 2.9 and a flow rate of 1.0 mL/min.

#### Enzymatic Hydrolysis of Racemic 1-Phenylethanol Acetate (*rac*-AFE)

The mobile phase consisted of acetonitrile (40%) and 10 mM ammonium phosphate buffer (60%) at pH 2.9 and a flow rate of 1.0 mL/min.

### 3.12. Determination of Enantiomeric Excess (ee)

At different degrees of conversion, the *ee* of the produced acid was analyzed by chiral reverse-phase HPLC. The column was a Chiracel OD-R, the mobile phase was an isocratic mixture of 5% acetonitrile and 95% ammonium phosphate 10 mM, at pH 2.3 and the analyzes were performed at a flow rate of 0.5 mL/min by recording the absorbance at 225 nm. Except by *rac*-MEMA, the mobile phase was an isocratic mixture of 30% acetonitrile and 70% ammonium phosphate 10 mM, pH 2.3.

### 3.13. Calculation of E (Enantiomeric Ratio)-Values

The *E*-value was calculated from the ratio between the enantiomers of both released acid. Conversion degree was always between 10% and 15%. The formula used was the one reported by Chen et al. [42]:

$$E=\ln\frac{\left[\left(1-X\right)\left(1-ee\right)\right]}{\left[\left(1-X\right)\left(1+ee\right)\right]}$$

where *X*: conversion; *ee*: enantiomeric excess.

### 3.14. Reproducibility of Experiments

All experiments were performed at least three times to confirm the results obtained and the standard.

## 4. Conclusions

The lipase from *H.* *pseudokoningii* was successfully immobilized in all the supports tested and besides, hyperactivation was attained with several of them. Best results were obtained when the lipase was covalently immobilized on glutaraldehyde-activated supports. Furthermore, lipase was highly stabilized in relation to temperature and the presence of denaturating solvents. The higher stabilization was attained with cyclohexane as solvent. Derivatives also showed enough recycling capability, suggesting possible applicability in industrial processes aimed at the hydrolysis of fats and oils.

## Figures and Tables

**Table 1:** Immobilization on covalent supports.

Derivatives	Immobilization Yield (%)	Activity Recovery (%)
CNBr	100 ± 0.7	81 ± 0.5
GX	100 ± 0.5	14 ± 0.2
GA	100 ± 1.0	135 ± 0.6
GA-crosslink	100 ± 0.9	236 ± 0.5

**Table 2:** Hydrolysis of sardine oil catalyzed by lipase derivatives.

Derivative	Activity ^a^	Selectivity ^b^
CNBr	18 ± 1.0	3 ± 0.5
GX	22 ± 1.1	4 ± 0.3
GA	28 ± 0.8	7 ± 0.1
GA-crosslink	27 ± 0.9	6 ± 0.5

^a^ Activity is expressed as nmol of polyunsaturated fatty acids (PUFAS —EPA and DHA—released per minute and per gram of the immobilized enzyme; ^b^ Selectivity is expressed as the ratio between released EPA and DHA. Selectivity was measured at the first stages of hydrolysis.

**Table 3:** Hydrolysis of açaí, cotton seed and grape seed oils catalyzed by the derivatives of *H. pseudokoningii* lipase.

Derivative	Activity on Açaí Oil Total U	Activity on Cotton Seed Oil Total U	Activity on Grape Seed Oil Total U
CNBr	367 ± 12	1083 ± 14	833 ± 12
GX	200 ± 10	167 ± 11	83 ± 19
GA	533 ± 7	283 ± 8	833 ± 9
GA-crosslink	283 ± 10	833 ± 18	1083 ± 12

Experiments were carried out as described in Methods.

**Table 4:** Activity and enantioselectivity of lipase derivatives in the hydrolysis of *racemic*-2-hydroxy-4-phenylbutanoic acid ethyl ester.

Derivative	SA	*E*	*ee*	EP
CNBr	7.41	3	2.6	*S*
GX	21	5	27.2	*R*
GA	13.33	3	6.4	*R*
GA-crosslink	21.88	5	34.3	*R*

Experiments were carried out as described in the Material and Methods section. SA—Specific activity—μmol X min^−1^ X mg prot^−1^; *ee*—Enantiomeric excess calculated by HPLC as described in the Materials and Methods section; *E*—Enantiomeric ratio as defined by Chen et al. [42]; EP—Enantiomeric preference.

**Table 5:** Activity and enantioselectivity of lipase derivatives in the hydrolysis of *racemic*-Butyryl-2-phenylacetic acid.

Derivative	SA	*E*	*ee* (%)	EP
CNBr	0.06	3	3	*S*
GX	5.23	4	12.3	*S*
GA	7.69	5	11.1	*R*
GA-crosslink	2.36	5	10.2	*R*

Experiments were carried out as described in the Materials and Methods section; SA—Specific activity—μmol X min^−1^ X mg prot^−1^; *ee*—enantiomeric excess calculated by HPLC as described in the Material and Methods section; *E*—Enantiomeric ratio as defined by Chen et al. [42]; EP—Enantiomeric preference.

**Table 6:** Activity and enantioselectivity of lipase derivatives in the hydrolysis of racemic-Methyl mandelate.

Derivative	SA	*E*	*ee* (%)	EP
CNBr	1.53	8	50	*S*
GX	1.85	14	66	*S*
GA	2.33	16	70	*S*
GA-crosslink	2.46	10	56	*S*

Experiments were carried out as described in the Material and Methods section; SA—Specific activity—μmol X min^−1^ X mg prot^−1^; *ee*—enantiomeric excess calculated by HPLC as described in the Material and Methods section; *E*—Enantiomeric ratio as defined by Chen et al. [42]; EP—Enantiomeric preference.

**Table 7:** Activity and enantioselectivity of lipase derivatives in the hydrolysis of *racemic*-1-phenylethanol acetate.

Derivative	SA	*E*	*ee* (%)	EP
CNBr	5.9	4	4.6	*R*
GX	3.1	4	1.3	*S*
GA	5.3	3	8.7	*S*
GA-crosslink	7.3	3	1.6	*S*

Experiments were carried out as described in the Material and Methods section; SA—Specific activity—μmol X min^−1^ X mg prot^−1^; *ee*—enantiomeric excess calculated by HPLC as described in the Material and Methods section; *E*—Enantiomeric ratio as defined by Chen et al. [42]; EP—Enantiomeric preference.
